# Biocidal effects of stem bark extract of *Chrysophyllum albidium* G. Don on vancomycin-resistant *Staphylococcus aureus*

**DOI:** 10.1186/s12906-016-1080-6

**Published:** 2016-03-22

**Authors:** David A. Akinpelu, Joseph O. Odewade, Olayinka A. Aiyegoro, Anofi O. T. Ashafa, Oluseun F. Akinpelu, Mayowa O. Agunbiade

**Affiliations:** Department of Microbiology, Obafemi Awolowo University, Ile Ife, Osun State Nigeria; GI Microbiology and Biotechnology Unit, Agricultural Research Council, Animal Production Institute, Irene, Pretoria 0062 South Africa; Plant Science Department, University of the Free State, Qwaqwa Campus, South Africa; Department of Biological Science, Faculty of Agriculture and Biotechnology, North West University, Mahikeng Campus, Mmabatho, 2735 South Africa

**Keywords:** *Chrysophyllum albidum*, Vancomycin resistant *Staphylococcus aureus*, Clinical samples, Methanolic extract, Phytochemicals, Minimum inhibitory concentrations, Minimum bactericidal concentrations, Time kill, Potassium ion, Nucleotide

## Abstract

**Background:**

*Staphylococcus aureus* causes variety of infections in humans and animals worldwide and predominates in surgical wound infections. This study assessed the antimicrobial potential of the stem bark extract of *Chrysophyllum albidum* against an array of vancomycin resistant *Staphylococcus aureus* (VRSA) isolated from clinical samples.

**Methods:**

The methanolic crude extract of the plant was preliminary screened for the presence of phytochemicals; after then, the extract was partitioned into n-hexane, chloroform, ethyl acetate and butanol fractions. A range of concentrations of the plant extract fractions was prepared to assess its antimicrobial potency; the minimum inhibitory concentrations (MICs); the minimum bactericidal concentrations (MBCs); the rate of killing; the potassium ion leakage potential and nucleotides leakage ability against the VRSAs.

**Results:**

The phytochemical screening revealed the presence of tannins, alkaloids, flavonoids, saponins, steroids, reducing sugars and terpenoids as major phytoconstituents resident in the crude plant extract. The two active fractions (n-hexane and butanol) at a concentration of 10 mg/ml exhibited antibacterial activities with the MIC and MBC values for the fractions ranged between 0.63–10 mg/ml and 1.25–10 mg/ml respectively. The time kill assay revealed that the antibacterial action of the two fractions are time and concentration dependent; the n-hexane and butanol fractions achieved 100 % kill on the test isolates at a concentration of 3 × MIC and 2 × MIC respectively after 120 min of reaction time. Varying amount of potassium ions as well as nucleotides were leaked from the test cells by n-hexane and butanol fractions.

**Conclusions:**

This study has established the possibility of developing antimicrobial agents of natural origin to manage possible infection from vancomycin resistant *Staphylococcus aureus* that are now developing multi-resistance against many antibiotics.

## Background

*Staphylococcus aureus* causes variety of infections in humans and animals worldwide and predominates in surgical wound infections [1]. This organism causes superficial skin infection and are also responsible for some life-threatening diseases such as sepsis, respiratory tract and bloodstream infections [2, 3]. The emergence of vancomycin resistant *Staphylococcus aureus* is a big concern that is threatening human’s health globally and has led to an increase in morbidity and mortality. The abrupt and continued increase in the use of vancomycin worldwide resulted in the increasing frequency of VRSA infections in hospitals [4]. The diseases caused by these multiple-drug resistant microorganisms are associated with prolonged hospitalization [5].

Methicillin a derivative of penicillin was used to treat infection caused by *S. aureus* after its development of resistant to penicillin but resistant to methicillin finally emerged in 1962 [6, 7]. Vancomycin was then considered the drug of choice for the treatment of infections caused by *S. aureus* for many years before the emergence of VRSA [8]. Emergence of clinical infection due to *S. aureus* in a patient in USA with high resistance to vancomycin was reported in 2002 [9]. Thus, there is an urgent need to source for antimicrobial agents especially of natural origin to combat the activities of these pathogens. Emergence of vancomycin resistance in *S. aureus* is a global issue, and thus, this study focused on the potency of stem bark extract of *Chrysophyllum albidium* on vancomycin resistant *S. aureus* isolated from clinical samples.

*Chrysophyllum albidium* belongs to the family sapotaceae. It is commonly called *Agbalumo* in Yoruba, *Udala* in Igbo and *Agbaluba* in Hausa languages. This plant is also referred to as “The white star apple” [10]. *Chrysophyllum albidium* is a popular tropical fruit tree and widely distributed in the low land rain forest zones [11].

In Nigeria this plant is distributed within the south-western part of the country [12]. *Chrysophyllum albidium* is rich in natural antioxidants which promote health [13]. This plant is highly rich in flavonoids, steroids, glycosides and saponins and thus serves as a source of anti-inflammatory, anti-spasmodic, as well as possesses diuretic properties [14]. Extracts from seeds and roots of *C. albidium* effectively arrested bleeding from fresh wounds and also promote wound healing [15]. *Chrysophyllum albidium* leaves are used as emollients and for the treatment of skin eruptions, diarrhoea and stomach ache [16]. The stem bark of this plant is used in preparation of decoction for the treatment of fever and black coated tongues caused by increase population of bacteria and yeast in the mouth [17]. Leaves extract from *C. albidium* exhibited antibacterial activities against *S. aureus*, *Escherichia coli*, *Salmonella typhi* and *Shigella* species [18]. Seeds and root extracts obtained from *C. albidium* exhibited anti-inflammatory, anti-diarrhoeal and anti-haemorrhoidal [14]. Onyeka [19] demonstrated hypoglycaemic, antioxidant and hepatoprotective activities of root bark extract of *C. albidium* in alloxan-induced diabetic rats.

## Methods

### Cultures of clinical strains of vancomycin-resistant *Staphylococcus aureus* and standard cultures of *Staphylococcus aureus*

Various clinical strains of VRSA used for this study were collected from stock collection of Microbiology Laboratory of both Obafemi Awolowo University Teaching Hospital, Ile Ife, Osun State, Nigeria and University College Hospital, Ibadan, Oyo State, Nigeria. These isolates were first sub cultured on mannitol salt agar medium (Biolab) to confirm their purity and strains before use. The isolates were confirmed to be true VRSA by subjecting them to susceptibility testing against vancomycin (Duchefa). The standard strains of *Staphylococcus aureus* of National Collection of Industrial Bacteria (NCIB) and American Typed Culture Collection (ATCC) were obtained from culture collection of Prof David Akinpelu, Department of Microbiology, Obafemi Awolowo University, Ile Ife, Osun State, Nigeria.

The inoculum of the test isolates were prepared using the colony suspension method as described by European Committee for Antimicrobial Susceptibility Testing (EUCAST) (2000). The inoculum were standardized using 0.5 McFarland standards.

### Preparation of plant materials and extraction

Fresh stem bark of *Chrysophyllum albidium* used for this study was collected from Opa Village, Ile Ife (7.4667° N, 4.5667° E), Osun State, Nigeria in April, 2014. The plant sample was identified in Herbarium of Department of Botany, Obafemi Awolowo University, Ile Ife, Osun State, Nigeria. Voucher specimen of the plant sample was prepared (Voucher Number IFE-17419) and deposited for reference purposes. The plant sample was air-dried until a constant weight was obtained. The dried stem bark was ground into powder using a mill (CHRISTY LABMILL, Christy and Norris Ltd; Process Engineers, Chelmsford, England), the powdered material was stored in an air-tight container for further use.

Exactly 1500 g of powdered sample was soaked in solution of methanol and sterile distilled water (3:2) (*v/v*) for 96 h. The mixture was regularly agitated throughout the period. The mixture was filtered into a clean flask and the filtrate collected was concentrated *in vacuo* in rotary evaporator to expel the methanol from the aliquot. The aqueous part collected was lyophilized to obtain crude extract of the plant. The yield was noted and has dark brown colour.

### Test for phytochemical compounds present in *C. albidum* extract

The phytochemical analysis of the extract was done using Trease and Evans [20] and Harborne [21] methods. The test include determination of the presence of tannins, alkaloids, flavonoids, saponins, steroids, reducing sugars and terpenoids in the extract.

### Fractionation of the crude extract from *C. albidium* stem bark

The crude extract was fractionalized using different organic solvents in order of their polarity. The solvent used are n-hexane, chloroform, ethyl acetate and n-butanol in that order. The yield obtained for each fraction was noted and kept in an air-tight container for further use.

### The antibiograms of the fractions obtained from the crude extract against the test isolates

The antibiogram of the fractions was determined using Akinpelu and Kolawole method [22]. The test isolates were re-activated in nutrient broth for 18 h before use. Exactly 0.1 mL of the standardized test isolates was transferred into molten Mueller-Hinton agar medium at 40 °C, thoroughly mixed and then poured into a sterile Petri dish. The plates were allowed to set and wells were bored into the medium with the aid of a sterile 6 mm cork borer. These wells were then filled up with the suspension of the fractions at a concentration of 10 mg/mL. Streptomycin and ampicillin each at a concentration of 1 mg/mL were used as positive control, while sterile distilled water in separate wells were used as negative control. The plates were allowed to stand on the laboratory bench for 1 h to allow proper in-flow of the extract into the medium. The plates were later incubated at 37 °C for 24 h, after which they were observed for zones of inhibition which indicates susceptibility of the test isolates to the extract.

### The minimum inhibitory concentrations (MIC) of the fractions against the test isolates

The MIC of the fractions against the test isolates was determined using EUCAST [23] method. Dilution of the fractions ranging between 0.313 and 5.00 mg/mL were prepared and incorporated into molten nutrient agar at 45 °C and poured into sterile plates. The plates were allowed to set and left on the laboratory bench overnight to ascertain of non-contaminant in the prepared plates. The plates were then inoculated with the standardized inoculum of the test isolates by streaking across the plate surface. These plates were incubated aerobically at 37 °C for 48 h and observed for any growth. The MIC was taken as the lowest concentration of the extract that inhibited the growth of the test isolates.

### The minimum bactericidal concentration (MBC) of the fractions against the test isolates

The MBC of the fractions against the test isolates was determined using Spencer [24] and Okore [25] methods with some modifications. Samples were taken from plates with no visible growth in MIC assay and sub-cultured onto freshly prepared nutrient agar plates and incubated at 37 °C for 72 h. The MBC was taken as the concentration of the extract that did not show any bacterial growth on fresh nutrient agar plates.

### Determination of rate of kill of the test isolates by the fractions

The assay of the rate of kill of the test isolates was determined using Odenholt method [26] with some modification. Culture of the test isolates was first standardized to approximately 10^6^ cfu/mL before use. Exactly 0.5 mL of the standardized suspension of the culture was added to 4.5 mL of different concentrations of the fraction relative to MIC. These were held at room temperature over a period of 2 h to determine the killing rate. Exactly 0.5 mL of the standardized suspension of the culture was added to 4.5 mL of different concentrations of the fraction relative to MIC. These were held at room temperature over a period of 2 h to determine the killing rate. A volume of 0.5 mL of each suspension was withdrawn at time intervals and transferred to 4.5 mL of recovery medium containing 3 % “Tween 80” to shake off the effect of the extract carry-overs from the test isolates. The suspension was then serially diluted and plated for viable counts. The plates were incubated at 37 °C for 48 h before reading. Control plates containing the test cells without the extract were set up along with the experimental. The emergent bacterial colonies were counted and compared with the counts of the culture control.

### Determination of potassium ion leakage from the test isolates by the fractions

Exactly 50 mL of harvested and washed cells (OD_470nm_ = 1.5) were placed in a clean 100 mL beaker which was magnetically stirred. A volume (5 mL) of ionic strength adjustment buffer (ISAB; 18.37 g of tetraethylammonium chloride in deionized water and made up to 100 mL) was added to the beaker. This ensured that the background ionic strength of all solutions was kept constant. The potassium ion sensing electrode (Qualiprobe QSE 314, EDT Instruments Waldershare Park, Dover, UK) and its reference electrode (Qualiprobe double junction reference electrode E8092, EDT Instruments) were placed into the cell suspension. The potential difference (mV) derived by the electrodes was measured using a Whatmann PHA 220 pH/mV meter (Whatmann Maidstone, UK). Bacterial cells were treated with various concentrations of the fraction of the plant extract relative to the MIC. The potassium efflux from the cells in the suspension was measured at time intervals over 2 h as a potential difference in mV. These values were converted to concentrations of K^+^ ions (M) by reference to a conversion graph, which had been constructed earlier using KCl standard solutions. The concentration of K^+^ ions released was plotted against time.

### Determination of nucleotides leakage from the test isolates by the fractions

The leakage of nucleotides from the test cells was determined using Heipieper method [27]. Washed cells of 18 h old test isolates was standardized (approximately 10^6^ cfu/mL) and treated with different concentrations of the fractions relative to the MIC at various contact time intervals over 2 h. Each suspension was centrifuged at 10,000 rpm and decanted. Wavelength of the supernatant collected was determined at 260_nm_ to quantify the amount of nucleotide leaked by comparing with the standard curve already plotted (A range of concentration from 0.5 to 5 μM of dNTP mix (dA, dC, dG, dT) (INQABA^©^ Biotech, Pretoria, South Africa) was prepared in TE buffer and used to generate the standard curve). The blank constitute sterile distilled water inoculated with the test isolates.

### Statistics and data processing

All experiments were done in triplicates. Data was analysed for a 4 × 4 Latin square design with the statistical program using the GLM model (Statistical Analysis Systems, SAS Institute, Cary, NC, USA, 2001). Results were contrasted with negative and a positive control. The means of the values was compared using independent *t* test of significance (*p* < 0.05).

## Results

Five fractions were obtained from the crude extract of *C. albidium* and they are n-butanol, n-hexane, chloroform, ethyl acetate and aqueous fractions. Two of the fractions (n-butanol and n-hexane) exhibited antimicrobial activities against the test isolates while other fractions were not active against the test isolates of VRSA. The zones of inhibition exhibited by both n-butanol and n-hexane fractions against the test isolates ranged between 10 and 16 mm. The zone of inhibition exhibited by n-butanol against *S. aureus* (NCIB 8588) was 17 mm while that exhibited against *S. aureus* (ATCC 6538) was 19 mm. On the other hand, zones of inhibition observed for n-hexane fraction against *S. aureus* (NCIB 8588) was 16 mm and for *S. aureus* (ATCC 6538) was 18 mm (Table 1). Streptomycin and ampicillin inhibited 33 out of 37 and 27 out of 37 test isolate respectively. The zones of inhibition exhibited by streptomycin against the test isolates ranged between 14 and 24 mm. On the other hand, ampicillin exhibited between 12 and 23 mm zones of inhibition against the test isolates. Overall, the two active fractions compared favourably with the two standard antibiotics used as positive control for this study.

**Table 1:** Sensitivity patterns exhibited by fractions obtained from *Chrysophyllum albidum* against the bacterial isolates

	Zones of inhibition (mm)^a^						
Bacterial isolates	BUT (10 mg/ml)	AQU (10 mg/ml)	n-HEX (10 mg/ml)	CHL (10 mg/ml)	ETHYL (10 mg/ml)	STREP (1 mg/ml)	AMP (1 mg/ml)
VRSA 1	11 ± 1.00	0	12.00	0	0	19 ± 1.41	0
VRSA 2	13 ± 1.41	0	14 ± 0.71	0	0	14 ± 0.58	0
VRSA 3	15 ± 0.71	0	13 ± 1.41	0	0	20 ± 0.71	17.00
VRSA 4	14.00	0	13 ± 1.00	0	0	23 ± 1.00	18 ± 0.71
VRSA 5	15 ± 0.58	0	12 ± 0.58	0	0	17 ± 1.20	16 ± 1.41
VRSA 6	15 ± 1.41	0	13 ± 1.41	0	0	18 ± 1.41	18 ± 1.00
VRSA 7	12.00	0	15 ± 1.00	0	0	20.00	20 ± 1.41
VRSA 8	11 ± 0.56	0	10.00	0	0	18.00	0
VRSA 9	11 ± 0.71	0	13 ± 0.56	0	0	16 ± 0.71	23 ± 0.56
VRSA 10	13 ± 1.20	0	14 ± 0.71	0	0	0	18.00
VRSA 11	11 ± 1.41	0	11 ± 0.54	0	0	0	16 ± 0.58
VRSA 12	0	0	0	0	0	17.00	0
VRSA 13	12 ± 1.00	0	14 ± 1.20	0	0	15.00	13 ± 0.71
VRSA 14	10.00	0	11 ± 0.56	0	0	18.00	0
VRSA 15	0	0	0	0	0	16 ± 0.58	0
VRSA 16	11 ± 1.41	0	13 ± 1.00	0	0	19.00	22 ± 0.71
VRSA 17	14.00	0	16.00	0	0	21 ± 0.56	13 ± 0.52
VRSA 18	13 ± 0.52	0	15 ± 0.52	0	0	17.00	23.00
VRSA 19	13 ± 1.00	0	12.00	0	0	19.00	0
VRSA 20	13 ± 0.54	0	15 ± 0.54	0	0	0	18.00
VRSA 21	13 ± 1.00	0	14.00	0	0	20 ± 1.00	15 ± 1.20
VRSA 22	11 ± 0.71	0	13 ± 1.41	0	0	22.00	19 ± 0.54
VRSA 23	13 ± 1.41	0	12.00	0	0	21 ± 0.54	17 ± 1.00
VRSA 24	10.00	0	14 ± 0.71	0	0	23.00	20.00
VRSA 25	11 ± 0.56	0	10.00	0	0	21.00	16 ± 1.41
VRSA 26	12.00	0	16 ± 1.00	0	0	23 ± 1.20	0
VRSA 27	15 ± 1.20	0	15 ± 1.20	0	0	22.00	19.00
VRSA 28	13 ± 0.71	0	14.00	0	0	20.00	0
VRSA 29	12 ± 0.58	0	12 ± 0.71	0	0	0	13 ± 0.54
VRSA 30	14.00	0	15 ± 0.58	0	0	24.00	20.00
VRSA 31	14 ± 0.71	0	11 ± 1.41	0	0	21.00	15.00
VRSA 32	10.00	0	12 ± 1.00	0	0	19.00	16.00
VRSA 33	13 ± 0.54	0	12.00	0	0	20.00	0
VRSA 34	11 ± 1.00	0	12 ± 0.71	0	0	19 ± 0.71	20.00
VRSA 35	16.00	0	15 ± 0.56	0	0	16.00	12.00
VRSA 36	17 ± 0.71	0	16 ± 0.52	0	0	23 ± 0.58	21.00 ± 0.71
VRSA 37	19 ± 1.20	0	18 ± 1.00	0	0	24 ± 0.70	22 ± 1.00

Key: BUT = Butanol fraction, AQU = Aqueous fraction, n-HEX = n-Hexane Fraction, CHL = Chloroform fraction, ETHYL = Ethy1 acetate fraction, STREP = Streptomycin and AMP = Ampicillin, ^a^ = Mean of three replicates, VRSA = Vancomycin resistant *Staphylococcus aureus* (Clinical strains), VRSA 36 = *Staphylococcus aureus* (NCIB 8588), VRSA 37 = *Staphylococcus aureus* (ATCC 6538)

The MIC and MBC exhibited by both n-butanol and n-hexane fractions against the test isolates were also determined. The MIC exhibited by n-butanol fraction ranged between 0.63 and 5.00 mg/mL while that of n-hexane was between the same range with that of n-butanol. The minimum bactericidal effects showed against the test isolates by both fractions ranged between 1.25 and 10.00 mg/mL. From all indications both n-butanol and n-hexane fractions exhibited equal antimicrobial activities against the test isolates (Table 2).

**Table 2:** The minimum inhibitory and bactericidal concentrations exhibited by the butanol and n-hexane fractions against susceptible bacterial isolates

	Butanol fraction		n-Hexane fraction	
Bacterial isolates	MIC (mg/ml)	MBC (mg/ml)	MIC (mg/ml)	MBC (mg/ml)
VRSA 1	5.00	10.00	2.50	5.00
VRSA 2	ND	ND	5.00	10.00
VRSA 3	1.25	2.50	1.25	2.50
VRSA 4	1.25	2.50	1.25	2.50
VRSA 5	2.50	5.00	5.00	10.00
VRSA 6	1.25	2.50	0.63	1.25
VRSA 7	2.50	5.00	1.25	2.50
VRSA 8	ND	ND	5.00	10.00
VRSA 9	5.00	10.00	ND	ND
VRSA 10	5.00	10.00	1.25	2.50
VRSA 11	1.25	2.50	2.50	5.00
VRSA 12	ND	ND	ND	ND
VRSA 13	ND	ND	ND	ND
VRSA 14	5.00	10.00	ND	ND
VRSA 15	ND	ND	ND	ND
VRSA 16	1.25	2.50	1.25	2.50
VRSA 17	1.25	2.50	0.63	1.25
VRSA 18	ND	ND	1.25	2.50
VRSA 19	5.00	10.00	ND	ND
VRSA 20	5.00	10.00	2.50	5.00
VRSA 21	2.50	5.00	ND	ND
VRSA 22	ND	ND	ND	ND
VRSA 23	1.25	2.50	1.25	2.50
VRSA 24	1.25	2.50	0.63	1.25
VRSA 25	2.50	5.00	2.50	5.00
VRSA 26	1.25	2.50	1.25	2.50
VRSA 27	2.50	5.00	2.50	5.00
VRSA 28	1.25	2.50	0.63	1.25
VRSA 29	1.25	2.50	5.00	10.00
VRSA 30	2.50	5.00	5.00	10.00
VRSA 31	0.63	1.25	0.63	1.25
VRSA 32	1.25	2.50	2.50	5.00
VRSA 33	2.50	5.00	0.63	1.25
VRSA 34	1.25	2.50	1.25	2.50
VRSA 35	ND	ND	ND	ND
VRSA 36	0.63	1.25	1.25	2.50
VRSA 37	1.25	2.50	2.50	5.00

KEY: VRSA = Vancomycin resistant *Staphylococcus aureus* (Clinical strains), MIC = Minimum Inhibitory Concentration, MBC = Minimum Bactericidal Concentration, VRSA 36 = *Staphylococcus aureus* (NCIB 8588), VRSA 37 = *Staphylococcus aureus* (ATCC 6538), ND = Not Done

The phytochemical compounds present in the extract were also investigated. The extract revealed the presence of tannins, alkaloids, flavonoids, saponins, steroids, reducing sugars and terpenoids (Table 3).

**Table 3:** Preliminary phytochemical compounds obtained from the stem bark extract of *Chrysophyllum albidum*

Chemical test	Result
Tannins	Positive
Alkaloids	Positive
Flavonoids	Positive
Saponins	Positive
Steroids	Positive
Reducing sugars	Positive
Terpenoids	Positive

**Fig. 1:**
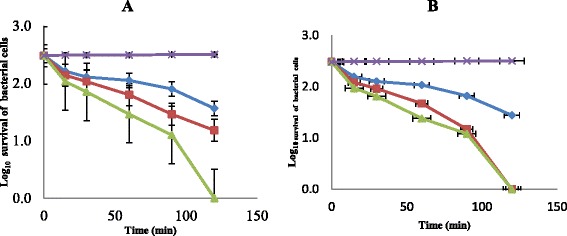
The extent and the rate of killing of test cells by n- hexane (**a**) and butanol (**b**) fractions at 1 × MIC (), 2 × MIC (), 3 × MIC () and control (). Each point represents the log_10_ of mean survival of bacterial cells at a particular time interval in the presence of the fraction

The bactericidal potentials of the two fractions were determined on the test isolates by assaying for the killing rate, potassium ion leakage and leakage of nucleotides. Figure 1a shows the extent and killing rate of the test isolates by n-hexane fraction. At a concentration of 1 × MIC after 15 min of contact time of the isolates with the fraction, 44.5 % of the test isolates was killed and this rose to 55.5 % at 30 min of contact time. At 60 min contact time the population of the test isolates killed was 61.3 % while the population rose to 73.0 % at 90 min contact time. Finally, at 120 min contact time 87.7 % of the test isolates were killed. This monophasic effects were also observed when the n-hexane fraction concentrations were increased to 2 × MIC and 3 × MIC. Finally 100 % cells killing was achieved at 3 × MIC concentration after 120 min of contact time of the test cells with the fraction (Fig. 1a). The same trend of reactions were observed when the test isolates were subjected to n-butanol fraction (Fig. 1b). The rate at which the test cells were killed increases with the concentrations as well as increase in contact time. This is also an indication of monophasic effects. Thus, the bactericidal effects of both n-hexane and n-butanol were through cell membrane disruption as indicated in the experiment.

**Fig. 2:**
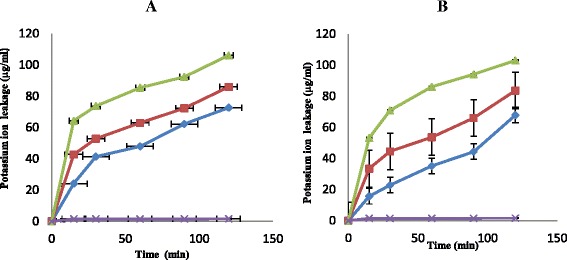
The effect of the n- hexane (**a**) and butanol (**b**) fractions on potassium ion leakage from test cells at 1 × MIC (), 2 × MIC (), 3 × MIC () and control (). Each point represents the amount of potassium ions leaked (μg/ml) from the cells at a particular time interval in the presence of the fraction

Figure 2a shows the effect of n-hexane fraction on potassium ion leakage from the test cells at concentrations of 1 × MIC, 2 × MIC and 3 × MIC. The quantity of potassium ion that got leaked out of the test cells at 1 × MIC after 15 min contact time of the cells with the fraction was 24.17 μg/mL. When the contact time reached 90 min at the same concentration, the quantity of potassium ion leaked out of the cells reached 62.23 μg/mL and about 72.63 μg/mL was observed at 120 min of contact time (Fig. 2a). This monophasic effects were also observed for n-butanol fraction reactions with the test cells (Fig. 2b). Increase in concentrations and contact time resulted in increase in percentage of the test cells killed. For example, at 1 × MIC and 120 min contact time the quantity of potassium ion leaked out of the test cells was 67.83 μg/mL. At concentrations of 2 × MIC and 3 × MIC and contact time of 120 min, the quantities of potassium ion leaked out of the test cells were 83.59 and 103.0 μg/mL respectively (Fig. 2b).

**Fig. 3:**
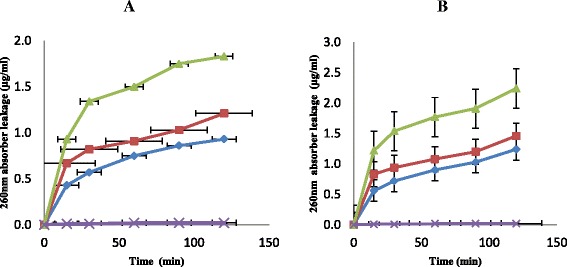
The effect of the n-hexane (**a**) and butanol (**b**) fractions on nucleotide leakage from test cells at 1 × MIC (), 2 × MIC (), 3 × MIC () and control (). Each point represents the quantity of nucleotides leaked (μg/ml) from the cells at a particular time interval in the presence of the fraction

The extent and damaged done to the test cells membrane was also observed through nucleotide leakage (Fig. 3). The two fractions exhibited appreciable nucleotide leakage as observed in the results. When the test cells were subjected the effects of n-hexane fraction at a concentration of 1 × MIC and at 15 min contact time the quantity of nucleotides leaked from the cells was 0.43 μg/mL. At 30 min contact time, the quantity leaked increased to 0.57 μg/mL and got to 0.75 μg/mL after 60 min of contact time. There was an increase in the quantity of nucleotides leaked out of the cells to 0.93 μg/mL when the contact time was increased to 120 min. The same trend of nucleotide leakage from the test cells was observed when the concentrations of n-hexane fraction were increased by 2 × MIC and 3 × MIC (Fig. 3a). The effects of n-butanol fraction against the test isolates was also observed as shown in Fig. 3b. There was an increase in the quantity of nucleotides leaked from the cells as the concentrations and contact time increased. For example, at 1 × MIC, 2 × MIC and 3 × MIC concentrations after 120 min of contact time, the quantities of nucleotides leaked from the cells were 1.03, 1.46 and 2.24 μg/mL respectively (Fig. 3b).

## Discussion

The antimicrobial potentials of stem bark extract of *Chrysophyllum albidium* were investigated on various isolates of vancomycin resistant *S. aureus* isolated from clinical samples along with two typed cultures, one of NCIB and the other of ATCC. *Staphylococcus aureus* is a pathogen and is responsible for various ailments in humans and animals. This organism has developed resistant to many antibiotics used as therapy against infections caused by it. Methicillin was later chosen as a drug of choice for the treatment of infections caused by this pathogen but later developed resistant to it [6, 7]. Later, vancomycin was adopted to combat the activities of *S. aureus* and this drug is no longer showing potency towards the treatment of infection caused by this pathogen [8]. The results obtained from our studies on fractions obtained from *C. albidium* stem bark extract showed that the extract exhibited high antibacterial activities towards various isolates of vancomycin resistant *S. aureus* used for this studies. Fractions obtained from this extract compared favourably with the two standard antibiotics–ampicillin and streptomycin used as positive controls. This is an indication that *C. albidium* stem bark extract could serve as a remedy to combat the infections caused by VRSA along with other pathogens that have now developed resistance to various antibiotics now in circulation used as therapy against infections caused by microorganisms. *Chrysophyllum albidium* is used among many tribes in West Africa for the treatment of various infections caused by microorganisms. Extracts obtained from *C. albidium* exhibited the presence of phytochemicals which include flavonoids, tannins, saponins, alkaloids, steroids, reducing sugars and terpenoids. These phytochemicals contributed to the bioactive potentials of this plant. Phytochemicals have been reported to be biologically active [19] and thus contributed to the antimicrobial activities of this plant. These facts supported the usefulness of *C. albidium* in folklore remedies. The natural products and phytochemical compounds present in this plant can be exploited for the development of novel bioactive compounds for the treatment of infections caused by VRSA and other pathogens that are now becoming resistant to antibiotics.

Mode of action of n-hexane and n-butanol fractions from crude extract of *C. albidium* were also investigated through rate of killing, leakages of potassium ions and nucleotides from the test cells. *Chrysophyllum albidium* extract exerted a biocidal effects on the test cells and this might have been contributed by the phytochemicals present in this plant’s extract. The killing rate exhibited by both n-hexane and n-butanol showed a 100 % kill of the test cells at a concentration of 3 × MIC within the shortest time of 120 min of the contact time (Fig. 1). The ability of plant extract to eliminate or kill organisms at the shortest period of time is generally accepted definition of bactericidal activity in antibiotics [28]. These fractions also exhibited appreciable potassium ions and nucleotides leakage from the test cells which also led to the death of the organisms (Figs. 2 and 3). Leakage of potassium ions from bacterial cell could lead to deactivation of important enzymes required for cell metabolism and this effects could be lethal on the cells [29]. Potassium is the major monovalent intracellular cations in cells, and its uptake is essential for all living organisms [30]. Potassium has many key functions within bacterial cells and this include activation of intracellular enzymes. It also act as an intracellular second messenger and involved in the maintenance of a constant internal pH and membrane potential [31]. In addition, potassium plays an important function as an osmotic solute. Potassium ions transport is a critical determinant of growth and survival through its role in regulating cytoplasmic pH and cell turgor [32, 33]. Thus leakage of potassium ions from bacterial cells will have a serious effect on such cells as this leads to their death. Leakage of intracellular materials from the cells is an indication of damage to the cell membrane. Thus permeability of the cytoplasmic membrane will lead to the loss of cellular matters and consequently results in cell death [32]. From our results in this study, *C. albidium* extract might have caused disruption in cell membrane of VRSA used for this study and thus led to their death. This observation was also noted by [33]) in their work on *Candida albicans* and *C. krusei*.

## Conclusion

The bactericidal activities exhibited by *C. albidium* extract against VRSA used in this study revealed a significant therapeutic potential of this plant and supported its usefulness in folklore remedies for the management of infections caused by pathogens. The ability of the plant extract fractions obtained from *C. albidium* to kill VRSA in this study at low concentration and minimal contact time has established the potential of the plant as a template for future drugs that could be formulated to combat infections caused by VRSA; such drug would be useful in combating the menace of VRSAs in human and animal health.
